# Double Duct to Mucosa Pancreaticojejunostomy for Bifid Pancreatic Duct following Pylorus Preserving Pancreaticoduodenectomy: A Case Report

**DOI:** 10.1155/2012/657071

**Published:** 2012-12-05

**Authors:** K. Vasiliadis, C. Papavasiliou, N. Lamprou, P. Delivorias, S. Papaioannou, A. Karagiannidis, C. Makridis

**Affiliations:** ^1^First Department of General Surgery, General Hospital Papageorgiou, West Ring Road, Nea Efkarpia 56 403, Thessaloniki, Greece; ^2^Department of Radiology, General Hospital Papageorgiou, West Ring Road, Nea Efkarpia 56 403, Thessaloniki, Greece; ^3^Department of Gastroenterology, Euromedica Kyanous Stavros General Hospital, Vizyis-Vyzantos 1, 54 636 Thessaloniki, Greece

## Abstract

Bifid pancreatic duct represents a relatively rare anatomical variation of the pancreatic ductal system, in which the main pancreatic duct is bifurcated along its length. This paper describes the challenging surgical management of a 68-year-old male patient, with presumptive diagnosis of periampullary malignancy who underwent a successful double duct to mucosa pancreaticojejunostomy for bifid pancreatic duct. Following pylorus preserving pancreaticoduodenectomy, careful intraoperative inspection of the cut surface of the residual dorsal pancreas identified the main in addition to the secondary pancreatic duct orifice. Bifid duct anatomy was confirmed via intraoperative probing and direct visualization of the ductal orifices. A decision was made for the performance of an end-to-site double duct to mucosa pancreaticojejunostomy. Postoperative outcome was favorable without any complications. Although bifid pancreatic duct is relatively rare, pancreatic surgeons should be aware of this anatomical variation and be familiar with the surgical techniques for its successful management. Lack of knowledge and surgical expertise for dealing with this anatomical variant may lead to serious, life threatening postoperative complications following pancreatic resections.

## 1. Introduction

Embryologically the pancreas develops by the fusion of dorsal and ventral pancreatic elements. The ventral pancreatic bud gives rise to part of the head and uncinate process, while the remainder of the head, body, and tail of the pancreas develops from the dorsal pancreatic bud [1]. These structures normally merge by the sixth-to-seventh week of gestation giving rise to the developed pancreas, which has normally a dominant ductal system. Initially, the ventral bud of the pancreas is bilobed. This configuration eventually regresses however, in some individuals a remnant may persist causing anatomical variants of the uncinate process branches and/or aberrant pancreatic tissue in the gut [2]. Similarly, the dorsal pancreatic element may be also bilobed, constituting the possible cause of developmental abnormalities of the dorsal pancreatic ductal system, such as bifid pancreatic duct [3].

Pylorus preserving pancreaticoduodenectomy (pp PD) entails exeresis of both the entire ventral and part of the dorsal pancreas. Following resection, the stump of the remaining dorsal pancreas, which usually contains a single main pancreatic duct, is anastomosed to the jejunum. When PD is indicated, the presence of a bifid pancreatic duct poses a surgical challenge because if not managed properly, it accounts for serious postoperative complications, such as obstructive pancreatitis or pancreatic leakage.

This paper describes the challenging surgical management of a patient with bifid pancreatic duct in whom a successful double duct to mucosa pancreaticojejunostomy was performed, following pylorus preserving pancreaticoduodenectomy.

## 2. Case Report

A 68-year-old male patient was referred to our surgical department with a recent history of relapsing episodes of acute pancreatitis, obstructive jaundice, postprandial low back pain, and nausea and weight loss (9 kg over the last 3 months). He had no symptoms associated with pancreatic endocrine or exocrine dysfunction, such as diabetes mellitus, diarrhea, or steatorrhea. He had no previous operations, nor did he smoke, overeat, or drink alcohol. His medical history included chronic atrial fibrillation for which he was under medication.

Physical examination on admission revealed malnutrition, scleral icterus, pruritus, and mild tachycardia (heart rate 90) with, however, a normal blood pressure. No acute distress was noted, while tenderness was palpated in the epigastrium in addition to a tense palpable gallbladder in the right hypochondrium. Laboratory analysis at the time of referral, showed normal white blood cell count, C-reactive protein 3.5 mg/dL, and serum pancreatic amylase level 34 U/l. Serum direct bilirubin (8.9 mg/Dl), aspartate (70 IU/l) and alanine aminotransferases (64 IU/l), alkaline phosphatase (410 IU/l), and *γ*-glutamyltransferase (74 IU/l) levels were also markedly elevated. Serum levels of CEA and Ca 19-9 were normal.

**Figure 1:**
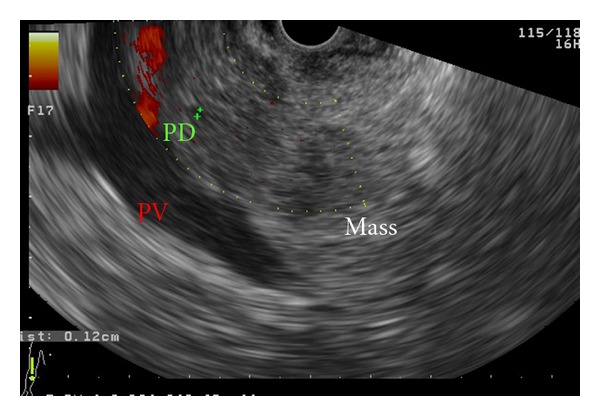
Linear EUS showing an inhomogeneous hypoechoic mass with irregular borders in the head of the pancreas. The echotexture of the pancreatic parenchyma appears heterogeneous with a coarse reticular pattern. A single pancreatic duct (PD) with a maximal diameter of 4 mm could be delineated in the head of the pancreas, appearing uniform with anechoic margins. A patent portal vein (PV) was also depicted bellow the PD with no evidence of tumor invasion.

**Figure 2:**
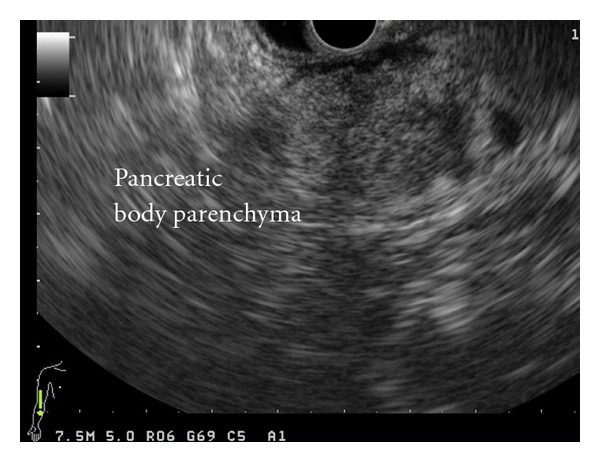
Linear EUS showing a markedly inhomogeneous pancreatic body parenchyma. The ultrasound wave was unable to delineate anatomical structures corresponding to either the main or the secondary pancreatic duct.

Computed tomography and magnetic resonance imaging studies (MRI-MRCP) identified a focally enlarged pancreatic head parenchyma and a common bile duct (CBD) stricture. Subsequent endoscopic ultrasound (EUS), with a linear echoendoscope, identified an inhomogeneous pancreatic head parenchyma in addition to an irregular hyperechogenous narrowing of the intrapancreatic part of the common bile duct, with dilatation above. The stenosed bile duct wall appeared thickened (>3 mm), while no abnormalities were noted in the pancreatic ductal system (Figures 1 and 2). EUS-fine needle aspiration (FNA) cytology from the bile duct stricture was negative for malignancy.

Endoscopic retrograde cholangiopancreatography (ERCP) followed that showed a dilated CBD (16 mm in diameter), which abruptly irregularly narrowed at the level of the head of the pancreas in addition to gallbladder debris. Because of technical difficulties a pancreatography was not obtained. During ERC a plastic stent was inserted to the common bile duct for jaundice relief. Although intraductal bile duct aspiration samples taken during ERC were negative for malignancy, given the cholangiographic findings a high index of suspicion was raised for a distal common bile duct malignancy. Therefore, taking also into account the patient's wish, he was elected to undergo operative exploration for the treatment of a presumptive periampullary malignancy and associated clinical symptoms.

**Scheme 1:**
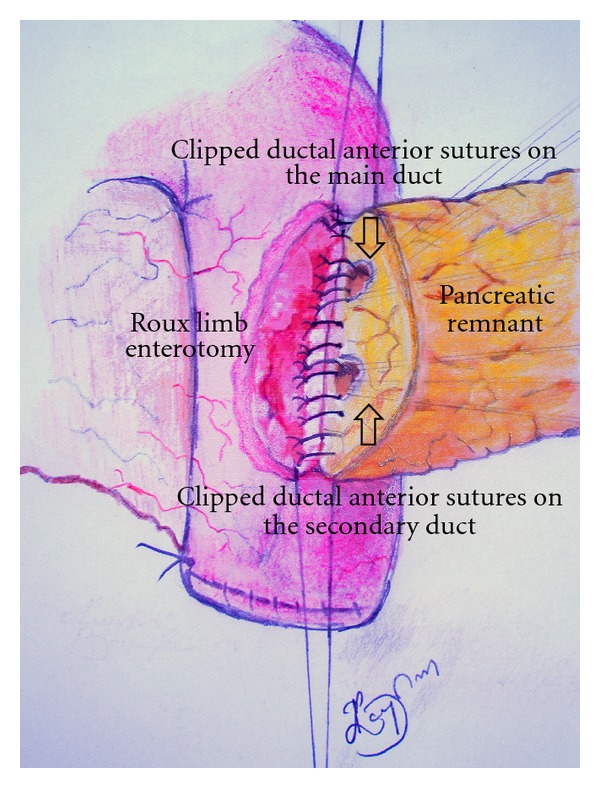
This scheme demonstrates the adaptation of the cut edge of the jejunum to the surface of the dorsal pancreatic remnant at the time of completion of the posterior inner row of the two-layer, single-stich (with monofilament absorbable, PDS 5-0; Johnsosn & Johnsosn with atraumatic JRB-1 needle), double duct to mucosa adaptation. Special attention was given to accomplish a duct to mucosa adaptation between duct epithelium and jejunal mucosa as demonstrated in the scheme. A ductal stent or drainage has not been used. The anterior ductal sutures on both ducts, which have been placed previously as first step of the anastomosis, were integrated in order, and each suture was clipped with a mosquito clamp.

At surgery there was no evidence of metastatic disease. The entire pancreas was very firm with distinct fullness and fibrosis appreciated in the head, being highly suspicious for a malignant process. Although intraoperative fine needle aspirates of demarcated areas of abnormal pancreatic tissue involving the intrapancreatic part of the common bile duct were negative for malignancy, given the intraoperative findings a radical procedure was decided in the form of pylorus-preserving PD (ppPD) with systemic lymphadenectomy and total mesopancreas excision. The pancreatic neck was copiously and meticulously separated from the portal and mesenteric veins and the pancreatic parenchyma was divided with a scalpel. Following resection, subsequent operative exploration of the cut surface of the residual dorsal pancreas identified the main duct in addition to a secondary pancreatic duct orifice (approximately 4 mm and 3 mm in diameter resp.). Bifid ductal anatomy was subsequently confirmed via intraoperative probing using blunt-tipped probes and careful direct visualization of the ductal orifices. Following confirmation of this extremely rare pancreatic duct duplication variant and taking into account the large diameter of the secondary duct, which was obviously draining a significant part of the remaining pancreatic parenchyma it was assumed that a possible ligation and sacrifice of the secondary duct could cause serious postoperative life-threatening pancreatic complications. Therefore, a decision was made for the performance of a challenging double duct to mucosa end to site pancreaticojejunal anastomosis. Pancreaticojejunal anastomosis was performed according to the technique used by the Büchler's surgical team of the University of Heidelberg [4], with the placement of four sutures on the main duct and three on the secondary, first anteriorly from the outside in and then posteriorly from the inside out. The ductal stitches were part of the posterior and anterior inner row of the fourth-row (double-layer) pancreaticojejunal anastomosis (Scheme 1). While tying the knots care was taken to succeed a duct to mucosa adaptation between duct epithelium and jejunal mucosa. Postoperative outcome was favorable without any complications.

Histopathological examination of the resected specimen revealed chronic pancreatitis with diffuse parenchymal fibrosis. The pancreatic ducts were surrounded by fibrous inflammatory tissue. The intrapancreatic CBD showed similar histopathological changes resulting in a benign inflammatory stricture. There was also a regional lymphadenitis present but there was no evidence of lymphocytic infiltration of the pancreatic parenchyma.

**Figure 3:**
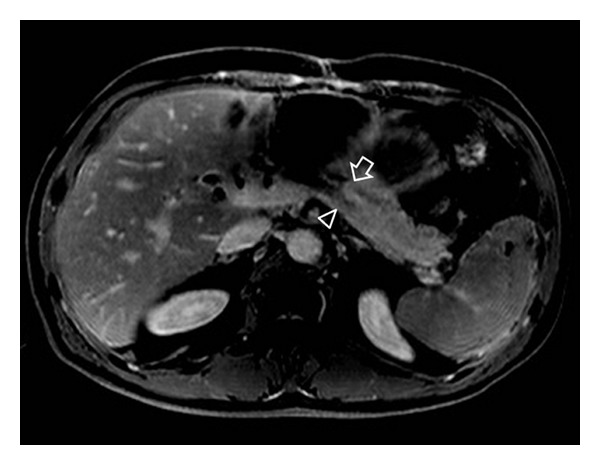
Postoperative followup dynamic magnetic resonance T1-weighted imaging of the remnant pancreas depicting the main as well as the secondary pancreatic duct (arrow and arrowhead resp.) draining the remaining dorsal pancreas at the level of the double pancreaticojejunal anastomosis.

The patient is doing well and has experienced no recurrent attacks of acute pancreatitis during a 12 months period of followup. Postoperative follow-up dynamic magnetic resonance imaging of the remnant pancreas depicted the main as well as the secondary pancreatic duct draining the remaining dorsal pancreas at the level of the double pancreaticojejunal anastomosis, allowing the confirmation of diagnosis of bifid pancreatic duct (Figure 3).

## 3. Discussion

Bifid pancreatic duct represents a relatively rare variation of the pancreatic ductal system and is categorized as a number variant of a duplication anomaly, in which the main pancreatic duct is bifurcated along its length [3]. Large series of endoscopic retrograde pancreatographies report a frequency of this anomaly ranging between 0.9% and 2.7% [5, 6].

The clinical significance of bifid pancreatic duct remains unsettled. In fact, while on one hand Bang et al. [5] and Uomo et al. [6] demonstrated no significant relationship between various pancreatic duct anomalies and pancreaticobiliary diseases or clinical conditions, on other hand Yatto and Siegel [7] and Krishnamurty et al. [8] claimed that the presence of bifid pancreatic duct alters the flow characteristics of pancreatic juice in the pancreatic ducts, thus increasing the risk of acute pancreatitis. The latter hypothesis could represent the underlying etiopathogenetic mechanism of relapsing episodes of acute pancreatitis in the present case.

Regardless of their clinical significance, anomalies of pancreatic ductal system, such as bifid pancreatic duct, could pose troublesome intraoperative technical difficulties during pancreatic resections [9–11]. This is because these anomalous pancreatic ducts and/or ductules are not expected to drain into the main pancreatic duct or into the jejunum in a pancreaticojejunal anastomosis and are considered as possible sites of pancreatic leakage. Therefore, it is extremely important to evaluate the pancreatic ductal system pre- and intraoperatively. In this case, preoperative imaging findings and especially EUS failed to provide information for the presence of a bifid pancreatic duct, making preoperative planning of the optimal treatment strategy impractical. Although EUS is accurate in ruling out pancreatic abnormalities, in this case EUS failed to delineate structures corresponding to either the main or the secondary pancreatic duct in the dorsal pancreas, probably because of the presence of a markedly inhomogeneous pancreatic parenchyma.

Instead, careful intraoperative inspection and direct visualization identified the main in addition to a secondary pancreatic duct on the cut surface of the residual dorsal pancreas. The bifid duct was subsequently confirmed via intraoperative probing, appearing too large in its drainage area to ligate and sacrifice. After establishing an intraoperative diagnosis and considering that ligation and sacrifice of the secondary duct could cause serious postoperative life-threatening pancreatic complications, a decision was made for the performance of a technically challenging double duct to mucosa end-to-site pancreaticojejunal anastomosis for the main and secondary duct, which proved a successful technique for managing such an anatomical variant.

The anastomotic technique used in the present case is actually a hybrid technique of the ivagination and duct to mucosa techniques, in which the entire cut surface of the pancreas is placed within the jejunum in an end-to-site fashion, thus allowing drainage of the main, variant and minor pancreatic ducts without using ductal stent or drainage catheter [12]. The main advantage of this technique is that it engages not only the main pancreatic duct but also all branch or variant pancreatic ducts located at the cut surface of the pancreas, thus preventing possible occurrence of leaks outside the anastomosis and the development of pancreatic fistula [13].

The present case underlines the importance of preoperative evaluation as well as intraoperative assessment of the pancreatic ductal system when performing PD. It also underlines a fairly common surgical dilemma facing pancreatic surgeons, that is, to operate or not patients with suspected but unproven periampullary malignancy, for which current data suggest that PD is an accepted surgical option for certain premalignant benign conditions such as chronic pancreatitis complicated with distal common bile duct stricture, especially when malignancy of the periampullary region cannot be definitively ruled out [14].

In conclusion, although bifid pancreatic duct is extremely rare, this case should alert clinicians to be aware of such an anatomical variant that may alter the flow characteristics in the pancreatic ductal system resulting in an increased risk of relapsing episodes of acute pancreatitis. Additionally, pancreatic surgeons should be aware of this entity and the surgical techniques for its successful management. The two-layer, single-stich double duct to mucosa adaptation pancreaticojejunostomy proved a successful technique for managing this rare anatomical entity. Lack of knowledge and surgical expertise for managing this anatomical variant may lead to serious, incurable postoperative pancreatic complications following pancreatic resections.
